# *Staphylococcus aureus* Keratitis in Taiwan: Genotyping, Antibiotic Susceptibility, and Clinical Features

**DOI:** 10.3390/ijms231911703

**Published:** 2022-10-03

**Authors:** Ching-Hsi Hsiao, Eugene Yu-Chuan Kang, Lung-Kun Yeh, David H. K. Ma, Hung-Chi Chen, Kuo-Hsuan Hung, Yhu-Chering Huang

**Affiliations:** 1 Department of Ophthalmology, Chang Gung Memorial Hospital, Linkou Medical Center, No. 5 Fu-Hsin Rd., Kweishan, Taoyuan 333, Taiwan; 2 College of Medicine, Chang Gung University, No. 261, Wenhua 1st Rd., Kweishan, Taoyuan 333, Taiwan; 3 Division of Pediatric Infectious Diseases, Department of Pediatrics, Chang Gung Memorial Hospital, Linkou Medical Center, No. 5 Fu-Hsin Rd., Kweishan, Taoyuan 333, Taiwan

**Keywords:** *Staphylococcus aureus*, methicillin-resistance *Staphylococcus aureus* (MRSA), community-associated (CA), Panton–Valentine leucocidin (PVL), keratitis

## Abstract

*Staphylococcus aureus* is an important pathogen for keratitis, a vision-threatening disease. We aimed to investigate the genotyping, antibiotic susceptibility, and clinical features of *S. aureus* keratitis, and to explore the possible role of Panton–Valentine leucocidin (PVL), a major virulence factor of *S. aureus.* We recruited 49 patients with culture-proven *S. aureus* keratitis between 2013 and 2017 at Chang Gung Memorial Hospital, Taiwan. PVL gene, multilocus sequence type (MLST), staphylococcal cassette chromosome mec (SCC*mec*), and pulsed-field gel electrophoresis (PFGE) were performed. Antibiotic susceptibility was verified using disk diffusion/E test. There were 49 patients with *S. aureus* keratitis; 17 (34.7%) were caused by methicillin-resistant *S. aureus* (MRSA) and 9 (18.4%) isolates had PVL genes. The predominant genotyping of MRSA isolates was CC59/PFGE type D/SCC*mec* V_T_/PVL (+). All methicillin-sensitive *S. aureus* (MSSA) and approximately 60% MRSA were susceptible to fluoroquinolones. No significant differences in clinical features, treatments, and visual outcomes were observed between MRSA/MSSA or PVL(+)/PVL(−) groups. In Taiwan, approximately one third of *S. aureus* keratitis was caused by MRSA, mainly community-associated MRSA. Although MRSA isolates were more resistant than MSSA, clinical characteristics were similar between two groups. Fluoroquinolones could be good empiric antibiotics for *S. aureus* keratitis.

## 1. Introduction

*Staphylococcus aureus* is a critical and commonly isolated human bacterial pathogen. Methicillin-resistant *Staphylococcus aureus* (MRSA), which is described as strains resistant to all available penicillin and other β-lactam antibiotics, warrants particular attention because of its limited treatment options. MRSA has been considered a hospital-acquired pathogen, but its prevalence in otherwise healthy patients without identified risk factors has reportedly increased. MRSA infections are classified as healthcare-associated MRSA (HA-MRSA) and community-associated MRSA (CA-MRSA); both strains are clinically, microbiologically, and genetically different [1,2,3]. In our 10-year (1999–2008) retrospective study, we found that the average rate of MRSA in *S. aureus* ocular infections was 52.8% [4]. The most common clinical presentation for both MRSA and methicillin-sensitive *S. aureus* (MSSA) ocular infections was keratitis [5]. We further analyzed clinical features and outcomes of *S. aureus* keratitis between 2006 and 2010, and found that advanced age and poor visual acuity at presentation, but not methicillin resistance, were important prognostic indicators for poor visual outcome in *S. aureus* keratitis [6,7]. However, our previously described studies were limited by lack of molecular data and the susceptibility of fluoroquinolones, the important ocular antibiotics.

The pathogenicity of *S. aureus* is related to various virulence factors, such as bacterial surface components, extracellular proteins, and toxins, which could cause profound tissue damage and modulate the immune response of the infections [8,9,10]. Among these virulence factors, Panton–Valentine leukocidin (PVL), which is predominantly present in CA-MRSA isolates but rarely observed in methicillin-sensitive *S. aureus* (MSSA) and HA-MRSA isolates, has caught the attention of numerous researchers [11,12,13,14,15]. PVL is a biocomponent toxin composed of LukS-PV and LukF-PV and assembles into a pore-forming heptamer on mainly polymorphonuclear neutrophils (PMN) and monocytes or macrophages, causing cell death; the toxin also induces the release of inflammatory cytokines from affected cells leading to tissue necrosis [16,17,18]. However, the role of PVL in the pathogenesis of *S. aureus* infections is equivocal, especially in vivo studies. Controversial data have been obtained using different animal models [12,13], which may have been caused by the differing immunology of the models, i.e., species specificity. Similarly, the effects of PVL on clinical presentation, severity, and outcomes are debated. Shalleross et al. recently presented results of a thorough systemic review of 76 clinical studies from 31 countries, and concluded that PVL genes are consistently associated with skin and soft-tissue infections and are comparatively rare in invasive disease but may increase morbidity in pediatric musculoskeletal infections [14]. However, little is known regarding the role of PVL genes in ocular infections compared with infections at other anatomical sites [19,20].

In this study, we aimed to investigate the molecular characterization, antibiotic susceptibility, and clinical features of *S. aureus* keratitis in Taiwan, and tried to determine the prevalence and clinical relevance of PVL in *S. aureus* keratitis. 

## 2. Results 

### 2.1. Molecular Typing of MRSA Isolates and PVL Presence

**Figure 1:**
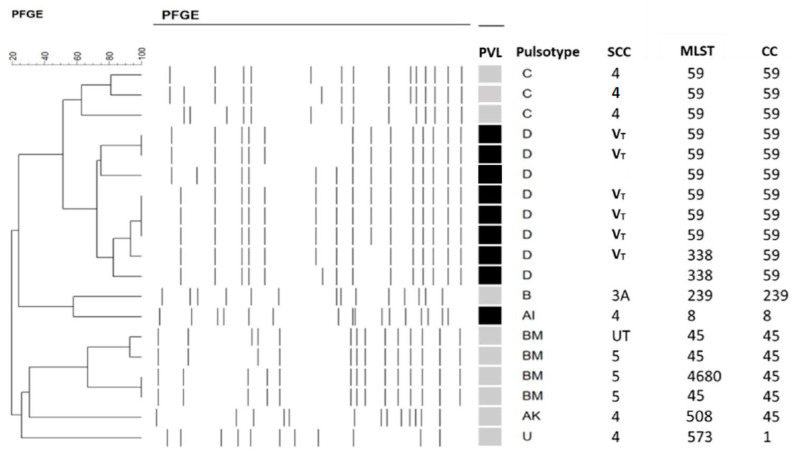
Molecular characterization of 17 MRSA isolates and two PVL(+) MSSA isolates. Black indicates PVL (+), and grey indicates PVL (−). MSSA: methicillin-sensitive *S. aureus*; MRSA: methicillin-resistant *S. aureus*; MLST: multilocus sequence type; PFGE: pulsed-field gel electrophoresis; PVL: Panton–Valentine leucocidin; SCC*mec*: staphylococcal cassette chromosome *mec*; UT: untypeable.

We identified 49 cases of *S. aureus* keratitis including 32 MSSA and 17 MRSA. Including MRSA (n = 7) and MSSA (n = 2), 9 of the 49 *S. aureus* isolates (18.4 %) were PVL (+). The molecular characterization of 17 MRSA isolates and two PVL (+) MSSA is displayed in Figure 1. Among the 9 PVL (+) *S. aureus* isolates, 8 belonged to clonal complex (CC) 59 including PFGE type D/SCC*mec* V_T_ (n = 6) and MSSA (without SCC*mec*, n = 2), and the other isolate was ST8/PFGE type A/SCC*mec* IV. The genotypes of the other 10 PVL (−) MRSA isolates included ST59/PFGE type C/SCC*mec* IV (n = 3), CC45/PFGE type BM [SCC*mec* V (n = 3), untypeable SCC (n = 1)], CC45/PFGE type AK/SCC*mec* IV (n = 1), ST573 (CC1)/PFGE type U/SCC*mec* IV (n = 1), and ST239/PFGE B1/SCC*mec* III (n = 1). Of the 17 (88.2%) MRSA isolates, 15 that carried type IV or V SCC*mec* belonged to CA-MRSA based on molecular definition. We did not report the genotyping of other MSSA isolates here because they had quite diverse molecular characteristics [21].

### 2.2. Antibiotics Susceptibility

As illustrated in Table 1, all *S. aureus* isolates were susceptible to vancomycin, teicoplanin, and tigecycline. A higher ratio of MSSA isolates was significantly more susceptible than MRSA to several antibiotics, including clindamycin (*p* = 0.009), erythromycin (*p* = 0.072), and all four fluoroquinolones (*p* = 0.001). When we further stratified *S. aureus* isolates by the presence of PVL gene, PVL (+) MRSA isolates seemed to be more susceptible to four fluroquinolones, but less susceptible to erythromycin and clindamycin than PVL (−) MRSA isolates, but no significant differences were noted.

### 2.3. Demographics and Predisposing Factors

Table 2 summarizes the demographics and risk factors related to *S. aureus* keratitis. No differences were observed in the distributions of age (*p* = 0.875), sex (*p* = 1.0), or source defined by epidemiology (*p* = 0.074) between MRSA and MSSA groups. Notably, 9 of 17 (52.9 %) patients with MRSA keratitis had no HA factors.

The most common predisposing factor for both MRSA and MSSA keratitis was ocular surface diseases and previous ocular surgery, which accounted for more than half of *S. aureus* keratitis. No differences in other local and systemic risk factors were discovered between MRSA and MSSA groups as well as PVL (+) and PVL (−) groups.

### 2.4. Clinical Findings

As shown in Table 3, no differences in ulcer location (*p* = 0.178), infiltration size (*p* = 0.284), or presence of hypopyon (*p* = 0.286) were identified between the MRSA and MSSA groups.

### 2.5. Treatment and Outcome

All patients with *S. aureus* keratitis were initially treated with topical levofloxacin (0.5%) or a combination of fortified antibiotics (vancomycin at 25 mg/mL and amikacin at 25 mg/mL). In 15 patients, the medication regimen was changed to 25 mg/mL vancomycin after obtaining the culture results. The rate of treatment modification did not differ significantly between the MRSA and MSSA groups (*p* = 1.0). Seven patients, three with MRSA and four with MSSA keratitis, required surgical interventions. Three patients, one with PVL (+) MRSA, the other with PVL (−) MRSA, and another with MSSA keratitis, received a patch graft with glycerol-preserved cornea for perforated ulceration. The other four patients (one PVL (+) MRSA and three MSSA keratitis) received amniotic membrane transplantation or tarsorrhaphy to promote re-epithelialization. No significant differences were identified in the rate of surgical intervention (*p* = 0.681), admission (*p* = 0.347), healing time (*p* = 0.648), or final visual outcome (*p* = 0.612) between the MRSA and MSSA groups (Table 4). There were no differences of treatments and outcomes between PVL (+) and PVL (−) *S. aureus* groups. Regression analysis demonstrated that a larger size of the corneal ulcer (*p* = 0.021), centrally located corneal ulcer (*p* = 0.038), elder age (*p* < 0.001), and higher LogMAR VA at presentation (*p* < 0.001) were associated with higher final LogMAR VA (i.e., worse vision). 

## 3. Discussion

This study updated the information regarding *S. aureus* keratitis and replenished the lack of genotyping and antibiotic susceptibility of fluoroquinolones in our previous study [6]. Our findings demonstrated that one third of *S. aureus* keratitis was caused by MRSA, around 90% of which belonged to molecular-defined CA-MRSA. The prevalence of the PVL gene was approximately one-fifth of *S. aureus* isolated from keratitis. The susceptibility of fluroquinolones was 100% and about 60% in MSSA and MRSA isolates, respectively. No significant differences in clinical features and outcomes were identified between MSSA and MRSA or PVL (+) and PVL (−) *S. aureus*.

In our previous 5-year (2006–2010) study of 59 patients with *S. aureus* keratitis, 26 infections were caused by MRSA, 12 (46.2%) of which had no healthcare factors, i.e., CA-MRSA by epidemiological definition [6]. In the current study, 9 of 17 (52.9%) MRSA isolates could be classified as CA-MRSA by epidemiological criteria (Table 2); however, 15 of 17 (88.2%) MRSA isolates belonged to CA-MRSA by molecular definition, but 6 had HA factors, which might imply the transmission of CA-MRSA into HA facilities. Other findings including antibiotic susceptibility and clinical features did not tend to change between these two study periods. 

The prevalence of CA-MRSA clone lineage differs by geographic area, but PVL and small SCC*mec* such as type IV or V are the most common elements in all major clones: ST8/SCC*mec* IV (USA300) in the United States [22], ST59/SCC*mec* V in Asia [23], ST93/SCC*mec* IV in Australia [24], and ST80/SCC*mec* IV in Europe [25]. In Taiwan, most CA-MRSA isolates share common molecular characteristics and belong to one of two major clones characterized by ST59/PFGE type C/SCC*mec* IV/PVL (−) (designated as Asian-Pacific clone) and ST59/PFGE type D/SCC*mec* V_T_/PVL (+) (designated as Taiwan clone) [26,27]. Recently, the ST45 strain has been identified in increasing frequency both in community and health-care settings in Taiwan [28]. which was also indicated in the current study.

In our study, nine PVL (+) isolates were identified: seven MRSA and two MSSA. The genotypes of six PVL (+) MRSA were CC59/PFGE type D/SCC*mec* V_T_, which belonged to the Taiwan clone of CA-MRSA. The genotype of the other PVL (+) MRSA was ST8/PFGE type A/SCC*mec* IV (i.e., USA 300). USA 300 is rare in Taiwan, accounting for 0.51% (27/5308 between 1995 and 2005) of MRSA [29]; the clone could have been imported from North America. The molecular characteristics of both PVL (+) MSSA isolates (CC59/PFGE type D) were similar to those of the dominant endemic CA-MRSA strain in Taiwan, which may corroborate the theory that CA-MRSA isolates are distinct strains, emerging de novo from MSSA isolates rather than from HA-MRSA isolates [17].

In the present study, MSSA isolates were susceptible to all tested antibiotics; it is no wonder that MRSA isolates were more resistant to certain antibiotics than MSSA. Notably, around 60% MRSA (even higher for PVL (+) MRSA) were susceptible to fluoroquinolones, which are the empiric antibiotics most frequently prescribed by ophthalmologists. Several studies have reported that the rate of in vitro resistance to fluoroquinolones has been increasing in MRSA keratitis [30,31,32]. Three national surveys of ocular isolates conducted in the United States reported that the rate of susceptibility to fluoroquinolones was only 15–25% for MRSA, whereas it was 80–90% for MSSA [33,34,35]. By contrast, 50–85.7% of MRSA and 100% MSSA isolates displayed susceptibility to fluoroquinolones in our study. Therefore, topical fluoroquinolones, the most popular commercially empiric antibiotics, could be a useful antibiotic for the treatment of *S. aureus* keratitis in Taiwan. If the infection does not respond well to fluroquinolones, fortified vancomycin, the last resort for MRSA, could be considered. 

In this study, we did not observe differences in clinical features and outcomes of *S. aureus* keratitis between the PVL (+) and PVL (−) groups, which contradicts the finding of a study by Sueke et al. [20]. They investigated 95 cases of *S. aureus* keratitis, nine (9.5%) of which were PVL (+) (eight MSSA and one MRSA); they reported nonsignificant trends for healing and treatment time, ulcers, and scar size, but they indicated that the overall clinical score and rate of surgical intervention were greater in the PVL (+) group than PVL (−) group with the effect being unrelated to MICs [20]. The discrepancy in results may have been caused by different dominant PVL (+) strains; CC59 CA-MRSA was dominant in our study, whereas MSSA was dominant in their study. Zaidi et al. used USA300, USA400, and isogenic PVL-deletion mutants to investigate the role of PVL in cultured human corneal epithelial cells and murine keratitis; they determined that PVL enhanced the virulence of a subset of MRSA strains (USA300) in a keratitis model but played an inconsistent role in pathogenesis and immunity to *S. aureus* corneal infection because of a strain-dependent effect [36]. It has been reported that the epidemiological association between invasive disease and PVL genes could be confounded by the extensive research conducted on USA300 [37]. Therefore, investigating other PVL (+) strains may clarify the role of PVL in the pathogenesis. As previously mentioned, two distinct CA-MRSA clones of ST59 are epidemic in Taiwan: the Asian-Pacific clone [PVL (−)] and the Taiwan clone [PVL (+)]. An epidemiological study of pediatric patients and an additional island-wide survey determined that severe infections were caused by the Taiwan clone [38,39], although the Asian-Pacific clone was more prevalent in colonizing healthy individuals [27,40]. In addition, a cultured PMN cytotoxicity study revealed that the supernatant of the Taiwan clone induced a greater degree of neutrophil lysis than that of the Asian-Pacific clone, and the extent of neutrophil lysis appeared to be correlated with the production of PVL in the Taiwan clone, which suggested that PVL is at least partially responsible for the cytotoxicity to neutrophils in ST59 CA-MRSA strains [41]. However, we did not observe an association between PVL and clinical features or outcomes in *S. aureus* keratitis in the current study. This discrepancy may have been caused by our comparing PVL (+) and PVL (−) isolates of all *S. aureus* keratitis, not CA-MRSA keratitis alone; however, further analysis of two ST59 CA-MRSA clones could not be performed because of the small sample size. In addition, the presence of PVL gene does not imply that the toxin is produced in vivo [42]. Moreover, apart from PVL, other bacterial and host factors may have contributed to the type and severity of clinical infections. 

The present study had some limitations. First, although we prospectively collected the isolates, we retrospectively reviewed the clinical data. Second, the relatively small sample might have affected the analysis of statistical significance. Third, in vitro susceptibility based on the serum systemic standards does not always correlate with clinical response, because no susceptibility standards for topical therapies have been established. Fourth, considering the different microbiological characteristics in differing geographic areas, the findings of the present study should not be generalized to other regions or populations. However, this study compensated for the lack of genotyping in our previous study [6,7] and provided the opportunity to explore the role of PVL in *S. aureus* keratitis in Taiwan. In addition, it opens perspectives for further experimental studies such as using enzymatic inhibitors assays, chromatographic characterization, comparative proteomic and gene expression mechanisms to compare PVL from different *S. aureus* strains.

In conclusion, our 5-year study determined that 34.7% (17/49) of *S. aureus* keratitis were caused by MRSA, and the prevalence of PVL gene in all *S. aureus* isolates from keratitis was 18.4% (9/49); CC59/PFGE type D/SCC*mec* V_T_/PVL (+) was the predominant clone. All MSSA isolates and more than 60% MRSA isolates were susceptible to fluoroquinolones. Ulcer location and size, advanced age, and poor VA at presentation were significantly associated with poor visual outcomes, whereas neither the presence of PVL nor methicillin resistance was a significant prognostic factor. This study provides the updated information including genotyping, antibiotic susceptibility, and clinical features of *S. aureus* keratitis, that may help clinicians to handle such patients.

## 4. Material and Methods

### 4.1. Study Population and Data Collection

*S. aureus* isolates were prospectively collected from patients with ocular infections in the microbiology laboratory of the Chang Gung Memorial Hospital, a 3700-bed medical center in Northern Taiwan, from January 1, 2013, to December 31, 2017. In total, 49 patients with *S. aureus* keratitis were identified. Medical records of these cases were retrospectively reviewed. Data collected included demographic information, medical and ocular history, systemic and local predisposing factors, clinical features, treatment, presenting and final visual acuity (VA), and length of follow-up. 

Healthcare-associated criteria were defined on the basis of the definition proposed by the Centers for Disease Control and Prevention Active Bacterial Core Surveillance sites [43] and were as follows: specimens obtained after 48 h of admission, history of hospitalization, surgery, dialysis, living in a long-term care facility within 1 year, permanent indwelling catheter. Cases with none of these features were defined as being community-associated.

Empirical or fortified antibiotics were administered hourly to treat *S. aureus* keratitis. The antibiotic treatment regimens were subsequently modified on the basis of culture results, susceptibility testing, and clinical response. Surgical interventions were indicated in some cases given the clinical conditions. The healing time was recorded once the infiltration had subsided and the epithelial defect healed. If data were available, VA was recorded at least 2 months after the keratitis had subsided and stabilized. Snellen VA values were converted into logMAR units for statistical analysis. VA—assessed through counting fingers, hand movements, light perception, and no light perception—was recorded as logMAR following the schedule suggested by Scott et al. [44].

### 4.2. Drug Susceptibility Tests

The antimicrobial susceptibility of all *S. aureus* isolates to antibiotics including cefoxitin, clindamycin, erythromycin, trimethoprim/sulfamethoxazole, teicoplanin, and vancomycin was routinely assessed using the disk diffusion method in our microbiology laboratory, following the guidelines of the Clinical and Laboratory Standards Institute antimicrobial susceptibility testing standards. Cefoxitin was used instead of oxacillin/methicillin to test for β-lactam antibiotic resistance. Moreover, an E-test (BioMerieux SA, Marcy-I’Etoile, France) was used to determine the susceptibility of isolates to fluoroquinolones—including ciprofloxacin, levofloxacin, gatifloxacin, and moxifloxacin—that were not included in the antibiotic susceptibility profiles in our microbiology laboratory.

### 4.3. Molecular Typing and Detection of PVL Gene

All *S. aureus* isolates were tested by the presence of PVL genes. The PCR amplification of the *lukS-PV* and *lukF-PV* genes encoding PVL components was described previously [11]. ENREF 6 Other molecular methods used in this study included pulsed-field gel electrophoresis (PFGE) with *Sma*I digestion, multilocus sequence typing (MLST), and SCC*mec* typing. PFGE was used to fingerprint the MRSA clinical isolates and PVL (+) MSSA according to the procedures described in a previous study [45]. The criteria proposed by Tenover et al. [46] were used to analyze the DNA fingerprints generated by the PFGE typing. The genotypes were designated in alphabetical order. PFGE patterns with -4-band differences from an existing genotype were defined as subtypes of that genotype and were labeled with Arabic number suffixes. MLST was performed as described elsewhere [47]; the allelic profiles were assigned through comparison of the sequences at each locus with those of the known alleles in the *S. aureus* MLST database (https://pubmlst.org/saureus/ (accessed on 30 August 2022)) and were defined as sequence types (STs) accordingly. The SCC*mec* typing was determined by a multiplex PCR strategy described previously [48]. SCC*mec* type V_T_ was determined by a particular primer described elsewhere [27]. Based on the molecular findings, the isolates carrying type I to III SCC*mec* and type IV or V SCC*mec* were classified as HA-MRSA and CA-MRSA, respectively [27].

### 4.4. Statistical Analysis

The continuous variables are presented as mean ± standard deviation, and the categorical variables are presented as the number of cases with a percentage. The intergroup differences in the variables were determined using Fisher’s exact test for analyzing nominal variables and the Mann–Whitney U test for continuous variables. Pearson correlation analysis was used to determine factors associated with visual outcome. Statistical significance was defined as *p* < 0.05.

## Figures and Tables

**Table 1:** Antibiotic susceptibilities of MRSA and MSSA isolates; both strains are further stratified by PVL gene.

Antibiotics	MRSA (n = 17) No.^a^ (%)	PVL (+) MRSA (n = 7) No. (%)	PVL (−) MRSA (n = 10) No. (%)	MSSA (n = 32) No. (%)	PVL (+) MSSA (n = 2) No. (%)	PVL (−) MSSA (n = 30) No. (%)
Clindamycin	7 (41.2)	1 (14.3)	6 (60.0)	32 (100)	2 (100)	30 (100)
Erythromycin	5 (29.4)	0 (0)	5 (50.0)	32 (100)	2 (100)	30 (100)
TMP-SMX	16 (94.1)	7 (100)	9 (90.0)	32 (100)	2 (100)	30 (100)
Teicoplanin	17 (100)	7 (100)	10 (100)	32 (100)	2 (100)	30 (100)
Tigecycline	17 (100)	7 (100)	10 (100)	32 (100)	2 (100)	30 (100)
Vancomycin	17 (100)	7 (100)	10 (100)	32 (100)	2 (100)	30 (100)
Ciprofloxacin	10 (58.8)	6 (85.7)	4 (40.0)	32 (100)	2 (100)	30 (100)
Levofloxacin	11 (64.7)	6 (85.7)	5 (50.0)	32 (100)	2 (100)	30 (100)
Gatifloxacin	11 (64.7)	6 (85.7)	5 (50.0)	32 (100)	2 (100)	30 (100)
Moxifloxacin	11 (64.7)	6 (85.7)	5 (50.0)	32 (100)	2 (100)	30 (100)

^a^ The number represents susceptibility to the antibiotics. MRSA: methicillin-resistant *Staphylococcus aureus*, MSSA: methicillin-sensitive *Staphylococcus aureus*, PVL: Panton–Valentine leukocidin; TMP-SMX: trimethoprim/sulfamethoxazole.

**Table 2:** Comparison of demographics and predisposing factors for MRSA and MSSA keratitis; both strains are further stratified by PVL gene.

Characteristics	MRSA	PVL (+) MRSA	PVL (−) MRSA	MSSA	PVL (+) MSSA	PVL (−) MSSA
	(n = 17)	(n = 7)	(n = 10)	(n = 32)	(n = 2)	(n = 30)
Age: median (range)	49 (2–93)	46 (8–93)	53 (2–88)	52 (6–88)	43.5 (18–69)	52 (6–88)
Sex: M/F	9/8	4/3	5/5	17/15	0/2	17/13
Community associated/	9 (52.9)/	4 (57.1)/	5 (50)/	25 (78.1)/	1 (50)/	24 (80)/
Healthcare-associated: n. (%)	8 (47.1)	3 (42.9)	5 (50)	7 (21.9)	1 (50)	6 (20)
Local factors: n (%) ^a^						
Contact lens wear	4 (23.5)	1 (14.3)	3 (30)	10 (31.3)	1 (50)	9 (30)
Trauma	1 (5.9)	0 (0)	1 (10)	3 (9.4)	0 (0)	3 (10)
Ocular surface disease	11 (64.7)	4 (57.1)	7 (70)	18 (56.3)	1 (50)	17 (56.7)
Previous ocular surgery	11 (64.7)	4 (57.1)	7 (70)	16 (50)	1 (50)	15 (50)
Usage of topical antibiotics/immunosuppressants	2 (11.8)	0 (0)	2 (20)	3 (9.4)	0 (0)	3 (10)
Systemic factors: n (%)						
Systemic comorbidities	8 (47.1)	3 (42.9)	5 (50)	11 (34.4)	1 (50)	10 (33.3)
Immunosuppressant	3 (17.6)	1 (14.3)	2 (20)	2 (6.3)	1 (50)	1 (3.3)
Systemic antibiotics	3 (17.6)	1 (14.3)	2 (20)	0 (0)	0 (0)	0 (0)

^a^ Total is greater than 100% because of some patients with multiple risk factors. F: female, M: male, MRSA: methicillin-resistant *Staphylococcus aureus*, MSSA: methicillin-sensitive *Staphylococcus aureus*, PVL: Panton–Valentine leucocidin.

**Table 3:** Clinical findings of MRSA and MSSA isolates; both strains are further stratified by PVL gene.

Clinical Findings	MRSA	PVL (+) MRSA	PVL (−) MRSA	MSSA	PVL (+) MSSA	PVL (−) MSSA
	(n = 17)	(n = 7)	(n = 10)	(n = 32)	(n = 2)	(n = 30)
Location: ^a^ No. (%)						
Central	9 (52.9)	4 (57.1)	5 (50)	13 (40.6)	1 (50)	12 (40)
Paracentral	7 (41.2)	2 (28.6)	5 (50)	10 (31.3)	1 (50)	9 (30)
Peripheral	1 (5.9)	1 (14.3)	0 (0)	9 (28.1)	0 (0)	9 (30)
Infiltration size (mm): No. (%)						
Small (<2)	7 (41.2)	3 (42.9)	4 (40)	19 (59.4)	0 (0)	19 (63.3)
Medium (2~6)	8 (47.1)	4 (57.1)	4 (40)	11 (34.4)	2 (100)	9 (30)
Large (>6)	2 (11.8)	0 (0)	2 (20)	2 (6.3)	0 (0)	2 (6.7)
Hypopyon	2 (11.8)	1 (14.3)	1 (10)	8 (25)	1 (50)	7 (23.3)

^a^ An ulcer was central if it encroached within 2 mm of fixation, it was peripheral if it involved a zone within 2 mm of the limbus, and paracentral if it was located between the central and peripheral zones. MRSA: methicillin-resistant *Staphylococcus aureus*, MSSA: methicillin-sensitive *Staphylococcus aureus*, PVL: Panton–Valentine leucocidin.

**Table 4:** Treatment and clinical outcome of MRSA and MSSA isolates; both strains are further stratified by PVL gene.

	MRSA	PVL (+) MRSA	PVL (−) MRSA	MSSA	PVL (+) MSSA	PVL (−) MSSA
	(n = 17)	(n = 7)	(n = 10)	(n = 32)	(n = 2)	(n = 30)
Modification of antibiotics: n (%)	5 (29.4)	3 (42.9)	2 (20)	10 (31.3)	1 (50)	9 (30)
Surgical intervention: n (%)	3 (17.6)	2 (28.6)	1 (10)	4 (12.5)	0 (0)	4 (13.3)
Admission: n (%)	6 (35.3)	4 (57.1)	2 (20)	15 (46.9)	2 (100)	13 (43.3)
Healing time ^a^ (days)	21.3 ± 33.0	17.0 ± 15.3	24.4 ± 42.6	15.1 ± 11.7	12.5 ± 0.7	16.4 ± 12.2
mean + standard deviation						
Healing time (days)/ulcer area median (range)	2.11 (0.12–30)	3.6 (1.5–4)	2 (0.12–30)	1.82 (0.4–20)	6.29 (0.58–12)	2.19 (0.4–20)
VA (LogMAR): median (range)						
At presentation	2.3 (0.2–3.2)	1.4 (0.2–3.2)	2.3 (0.2–3.2)	2.3 (0–3.2)	2.3 (2.3–2.3)	1.4 (0–3.2)
After treatment	2.3 (0.2–3.2)	1.5 (0.2–3.2)	2.3 (0.2–3.2)	1.5 (0–3.2)	1.4 (0.5–2.3)	1.0 (0–3.2)

^a^ Healing time: defined as the resolution of infiltration and epithelial defect. MRSA: methicillin-resistant *Staphylococcus aureus*, MSSA: methicillin-sensitive *Staphylococcus aureus*, PVL: Panton–Valentine leucocidin, VA: visual acuity.

## Data Availability

The data analyzed during this study are available on request from the corresponding author, Ching-Hsi Hsiao. The data are not publicly available due to it containing information that could compromise the privacy of research participants.
